# Gap Junction Coding Innexin in *Lymnaea stagnalis*: Sequence Analysis and Characterization in Tissues and the Central Nervous System

**DOI:** 10.3389/fnsyn.2020.00001

**Published:** 2020-02-25

**Authors:** Brittany A. Mersman, Sonia N. Jolly, Zhenguo Lin, Fenglian Xu

**Affiliations:** ^1^Department of Biology, College of Arts and Sciences, Saint Louis University, St. Louis, MO, United States; ^2^Henry and Amelia Nasrallah Center for Neuroscience, Saint Louis University, St. Louis, MO, United States

**Keywords:** innexin, gap junction, invertebrate, mollusk, gene specificity

## Abstract

Connections between neurons called synapses are the key components underlying all nervous system functions of animals and humans. However, important genetic information on the formation and plasticity of one type, the electrical (gap junction-mediated) synapse, is understudied in many invertebrates. In the present study, we set forth to identify and characterize the gap junction-encoding gene innexin in the central nervous system (CNS) of the mollusk pond snail *Lymnaea stagnalis*. With PCR, 3′ and 5′ RACE, and BLAST searches, we identified eight innexin genes in the *L. stagnalis* genome, named *Lst Inx1–Lst Inx8*. Phylogenetic analysis revealed that the *L. stagnalis* innexin genes originated from a single copy in the common ancestor of molluskan species by multiple gene duplication events and have been maintained in *L. stagnalis* since they were generated. The paralogous innexin genes demonstrate distinct expression patterns among tissues. In addition, one paralog, *Lst Inx1*, exhibits heterogeneity in cells and ganglia, suggesting the occurrence of functional diversification after gene duplication. These results introduce possibilities to study an intriguing potential relationship between innexin paralog expression and cell-specific functional outputs such as heterogenic ability to form channels and exhibit synapse plasticity. The *L. stagnalis* CNS contains large neurons and functionally defined networks for behaviors; with the introduction of *L. stagnalis* in the gap junction gene field, we are providing novel opportunities to combine genetic research with direct investigations of functional outcomes at the cellular, synaptic, and behavioral levels.

## Introduction

From simple reflexes to high cognitive functions including learning and memory, all nervous system operations rely on two main forms of synaptic communication to efficiently transmit signals: chemical (transmitter-mediated) and electrical (gap junction-mediated; Ovsepian, 2017). Gap junctions are formed by presynaptic and postsynaptic rafts of proteins that form intercellular channels, providing direct and efficient means of communication by allowing quick movement of ions and small molecules (<1 kDa) between the cytosol of coupled cells (Qu and Dahl, 2002). These gap junction-forming proteins have been identified in both vertebrates (named connexins) and invertebrates (named innexins: invertebrate analog of connexins) and exhibit structural homology despite their lack of sequence similarity. Originally thought to only have importance in invertebrates (Nagy et al., 2018), gap junctions are now known to be expressed throughout the mammalian nervous system and in various organs such as the heart, skin, kidney, eye, and inner ear (Dere and Zlomuzica, 2012). Mutations of gap junction-forming genes and associated proteins or dysfunction of gap junction activity are associated with many human diseases including cancer, deafness, and oculodentodigital dysplasia as well as fear-related behaviors and learning and memory deficiencies (Mas et al., 2004; Bissiere et al., 2011; Abrams and Scherer, 2012; Dere and Zlomuzica, 2012).

The discovery of direct intercellular communication was first made in the invertebrate crayfish (Furshpan and Potter, 1957, 1959) and then in lobster (Watanabe, 1958). Structural evidence of the existence of a “gap”-like nexus near plasma membranes of adjacent cells was revealed with electron microscopy studies in various tissues and cells in both vertebrates and invertebrates (Dewey and Barr, 1962, 1964; Farquhar and Palade, 1963), and molecular cloning and characterization of gap junction-forming genes were first made from tissues of humans and rats (Kumar and Gilula, 1986; Paul, 1986). The presence of innexin has been established in all invertebrates except for sponges and echinoderms (Watanabe, 1958; Skerrett and Williams, 2017); however, extensive studies of innexin genes encoding the gap junction-forming proteins have been severely restricted to a select few invertebrate model organisms, such as the fruit fly *Drosophila melanogaster*, the nematode *Caenorhabditis elegans*, and the medicinal leech *Hirudo verbana* (Phelan et al., 1998; Starich et al., 2001; Stebbings et al., 2002; Kandarian et al., 2012; Beyer and Berthoud, 2018). Such restrictions limit the extent to which evolutionary and functional analyses can be made; full genetic and molecular characterization of a gap junction system in a novel and easy-to-study invertebrate species is long overdue.

In this study, we introduce the freshwater pond snail *Lymnaea stagnalis* to the innexin gene field. Like its sea slug counterpart, *Aplysia californica*, *L. stagnalis* belongs to the phylum Mollusca and class Gastropoda. Mollusca is the second-largest phylum of invertebrate animals, and many mollusks, such as the gastropod *A. californica*, the cephalopod *Octopus vulgaris*, and the cephalopod squid *Loligo pealeii*, have proven to be valuable resources and models for making significant fundamental neurobiological discoveries (Tasaki and Takenaka, 1963; Brunelli et al., 1976; Tricarico et al., 2014). *L. stagnalis* has been used in studies ranging from simple locomotive behaviors (Syed and Winlow, 1991) to highly complex processes like synaptogenesis (Dmetrichuk et al., 2006) and learning and memory (Lukowiak et al., 2003; Kemenes and Benjamin, 2009; Marra et al., 2013). In addition, recent efforts have established a transcriptome (Feng et al., 2009; Sadamoto et al., 2012) and genome (Davison et al., 2016) assembly of *L. stagnalis*, making molecular and genetic research of the organism even more applicable and providing an invaluable tool for future work in the field of molecular neurobiology.

The *L. stagnalis* central nervous system (CNS) has been well described, and established neuronal networks are available, including morphological features, spatial topology, and types of synaptic connections (Winlow and Benjamin, 1976; Kemenes and Benjamin, 2009). Importantly, the *L. stagnalis* brain contains many gap junction-forming neurons that form well-defined networks for various behaviors. Table 1 summarizes several well-characterized and functionally defined electrical coupling neurons and networks in *L. stagnalis* CNS. For example, the pedal dorsal A (PeA) cluster neurons in the left and right pedal ganglia form gap junctions (electrical synapses) that control the cilia of the foot for locomotion (Syed et al., 1988; Kyriakides et al., 1989; Prinz and Fromherz, 2000; Xu et al., 2014). Similarly the cerebral A (CeA) cluster motoneurons in the left and right cerebral ganglia form gap junctions that control whole-body withdrawal response (Ferguson and Benjamin, 1991a). The two large, peptidergic neurons visceral dorsal 1 (VD1) and right parietal dorsal 2 (RPD2) form strong gap junction coupling that control *L. stagnalis* cardiorespiratory function (Benjamin and Winlow, 1981; Benjamin and Pilkington, 1986; Wildering et al., 1991a,b; Wildering and Janse, 1992; Ewadinger et al., 1994; Sidorov, 2012; Beekharry et al., 2015). In addition, many motor neurons and interneurons in the left and right buccal ganglia were shown to be electrically coupled to control feeding rhythm in *L. stagnalis* (Benjamin and Rose, 1979; Elliott and Kemenes, 1992; Ewadinger et al., 1994; Vehovszky and Elliott, 2001). The endocrine caudal dorsal cells (CDCs, comparable to the bag cells of *A. californica*) in the left and right cerebral ganglia fire synchronously and produce a prolonged afterdischarge, during which the ovulation hormone is released to promote egg-laying behavior (de Vlieger et al., 1980). Lastly, another type of neurosecretory neurons, the dark green cells mainly located in the left and right pleural ganglia and also in the left and right parietal and visceral ganglia, were found to form weak electrical coupling that regulates water and ion permeability through the skin for body osmolality control (Swindale and Benjamin, 1976; Benjamin, 1983). While well-defined neuronal networks including axon projections, synapse formation, and functional outcomes are known (see Table 1), most studies and knowledge of gap junctions in *L. stagnalis* and other species in the Mollusca phylum are limited to electrophysiological and behavioral work with little genetic information (Elliott and Benjamin, 1985; Carrow and Levitan, 1989; Ferguson and Benjamin, 1991a,b; Syed et al., 1991; Dargaei et al., 2014).

**Table 1:** Identified gap junction-forming neurons and networks in *L. stagnalis* nervous system.

Cell name	Ganglionic location	Function	Detection method	References
PeA/PeA	LPeG, RPeG	Locomotion	EP	Syed et al. (1988)
			EP	Kyriakides et al. (1989)
			EP	Prinz and Fromherz (2000)
			EP	Xu et al. (2014)
CeA/CeA	LCG, RCG	Whole-body withdrawal	EP	Ferguson and Benjamin (1991a)
VD1/RPD2	VG/RPG	Cardio-respiratory	EP	Benjamin and Winlow (1981)
			EP	Benjamin and Pilkington (1986)
			EP	Wildering et al. (1991a)
			EP	Wildering et al. (1991b)
			EP	Wildering and Janse (1992)
			Dye coupling	Ewadinger et al. (1994)
			EP	Sidorov (2012)
			EP	Beekharry et al. (2015)
Buccal neurons	LBG, RBG	Feeding	EP	Benjamin and Rose (1979)
			Dye coupling	Ewadinger et al. (1994)
			EP	Elliott and Kemenes (1992)
			EP	Vehovszky and Elliott (2000)
CDC	LCG, RCG	Hormone secretion during reproduction	EP, Horseradish peroxidase	de Vlieger et al. (1980)
Dark green cells	LPlG, RPlG, LPG, RPG, VG	Neurosecretory, ion and water regulation	EP	Benjamin (1983)
			Staining	Swindale and Benjamin (1976)

*Note: PeA, pedal dorsal A; CeA, cerebral cluster A, VD1, visceral dorsal 1; RPD2, right parietal dorsal 2; CDC, caudal dorsal cell; LPeG, left pedal ganglion; RPeG, right pedal ganglion; LCG, left cerebral ganglion; RCG, right cerebral ganglion; VG, visceral ganglion; RPG, right parietal ganglion; LBG, left buccal ganglion; RBG, right buccal ganglion; LPlG, left pleural ganglion; RPlG, right pleural ganglion; LPG, left parietal ganglion; EP, electrophysiology*.

To fill the hole in knowledge of the gap junction genes in *L. stagnalis*, we, for the first time, identified eight innexin genes named *Lst Inx1–Lst Inx8* (accession numbers are provided in Table 2). Phylogenetic analyses revealed the origin and evolutionary history of the eight paralogs in Mollusca. The expression pattern of one innexin, *Lst Inx1*, was analyzed *via* *in situ* hybridization (ISH) and demonstrated variable localization within ganglia that contain single cells known to form electrical synapses. Such information provides a necessary foundation for future investigation of the genetic and molecular mechanisms of nervous system development and function in *L. stagnalis* and other invertebrate species.

**Table 2:** Accession numbers of innexin genes in *L. stagnalis*.

Gene name	Accession number
*Lst Inx1*	MN480796
*Lst Inx2*	MN480797
*Lst Inx3*	MN480798
*Lst Inx4*	MN480799
*Lst Inx5*	MN480800
*Lst Inx6*	MN480801
*Lst Inx7*	MN480802
*Lst Inx8*	MN480803

## Materials and Methods

### Animals and CNS Dissection

The freshwater snails *L. stagnalis* were kept in artificial pond water at 20–22°C on a 12-h light/dark regimen and were fed romaine lettuce. Snails ~12 months old were used for innexin sequence identification, tissue expression, and ISH experiments, and snails 3–6 months old were used for cell culture and electrophysiological recordings. CNS isolation was performed as previously described (Syed et al., 1990). Briefly, snails were de-shelled and anesthetized in Listerine solution (21.9% ethanol and 0.042% methanol; department store; everywhere) diluted to 10% in *Lymnaea* saline (51.3 mM NaCl; 1.7 mM KCl; 4.0 mM CaCl_2_; 1.5 mM MgCl_2_, 10 mM HEPES, pH 7.9). Dissected central ring ganglia were used for cell culture, RNA/genomic DNA (gDNA) extraction, or ISH.

### Neuronal Cell Culture and Electrophysiological Recordings

*L. stagnalis* neuronal cell culture procedures and electrical coupling studies were described in detail in previous literature (Syed et al., 1990; Xu et al., 2014). Briefly, the dissected central ring ganglia were incubated in *Lymnaea* defined medium (DM; L-15; Invitrogen; special order; NaCl 40 mM, KCl 1.7 mM, CaCl_2_ 4.1 mM, MgCl_2_ 1.5 mM, HEPES 10 mM) containing 2 mg/ml of trypsin enzyme for 21 min and then transferred to 2 mg/ml of trypsin inhibitor solution for 15 min. The central ring ganglia were pinned down onto Sylgard-coated culture dishes containing high-osmolality DM solution where the outer connective tissues and inner sheathes were carefully removed using fine forceps. The gap junction-forming PeA neurons were isolated using a fire-polished glass pipette connected to a small syringe for creating negative (pull) and positive (push) pressure during cell pulling. Neurons were cultured in a soma–soma configuration in the presence of *Lymnaea* brain conditioned medium (CM) overnight. The next day, dual intracellular current-clamp recordings were made to verify functional electrical coupling between PeA neurons. Specifically, negative or positive currents were injected into one PeA cell (PeA-1) to induce a membrane potential change, and the consequent membrane potential change in the other cell (PeA-2) was monitored. If current injection-induced membrane potential changes in PeA-1 induced synchronized hyperpolarizing or depolarizing membrane potential changes in PeA-2, the formation of functional gap junctions was indicated.

### RNA and gDNA Extraction

RNA was extracted from the central ring ganglia and tissues of *L. stagnalis* with the RNeasy Mini Kit (Qiagen; 74104; Venlo, The Netherlands) according to the manufacturer’s instructions. An additional DNase digestion step (Qiagen; 79254; Venlo, The Netherlands) was added to the protocol to prevent DNA contamination. gDNA was used for normalization. gDNA extraction (Invitrogen; K1820-02; Carlsbad, CA, USA) was completed according to the manufacturer’s instructions.

### Identification of Innexin Genes in *L. stagnalis*

To determine whether homologs of innexin are present and expressed in *L. stagnalis*, we reverse-transcribed RNA extracted from whole CNS to cDNA with SuperScript II Reverse Transcriptase (Invitrogen; 18064-014; Carlsbad, CA, USA). PCR was then performed with an Eppendorf Mastercycler Gradient 5331 (Hauppauge, NY, USA), Taq DNA polymerase (New England Biolabs; M0273A; Ipswich, MA, USA), and degenerate primers designed for innexin detection in the crab *Cancer borealis* (Shruti et al., 2014): forward primer 5′-GAGGACGAGATCAA-GTACCACACATAYTAYCARTGG-3′ and reverse primer 5′-GGCATGAAGGTCAGGAA-GACGWRCCARAACC-3′. Because innexin genes contain regions rich in sequence conservation among all invertebrates (Beyer and Berthoud, 2018), it is interesting, but not surprising, that the degenerate primers designed for amplification in *C. borealis* also amplified a partial sequence in *L. stagnalis* (Supplementary Figure S1). The partial fragment was sequenced (Genewiz; South Plainfield, NJ, USA) and used to design primers (Table 3) for 3′ (Invitrogen; 18373-027; Carlsbad, CA, USA) and 5′ (Invitrogen; 18374-041; Carlsbad, CA, USA) rapid amplification of cDNA ends (RACE) to obtain a complete mRNA transcript from the start codon to the stop codon. We named the gene *Lst Inx1* (Table 2) according to common innexin naming strategies. We then used the translated amino acid sequence of *Lst Inx1* as a query in National Center for Biotechnology Information (NCBI) BLAST to search for its orthologous genes in other species. The top hits of the BLAST search were *A. californica pannexin1*, *Biomphalaria glabrata innexin unc-9 like*, and *Crassostrea gigas innexin unc-9*, which further supported that *Lst Inx1* belongs to the innexin gene family.

**Table 3:** Primers used for 3′ and 5′ RACE.

Paralog	3′ RACE gene specific primer (GSP)	3′ RACE nested GSP	5′ RACE GSP1	5′ RACE GSP2	5′ RACE nested GSP
*Lst Inx1*	5′-GGCACCTTTCTGACCGGG-3′	5′-CCGAGGTTCCCCAAGATCAC-3′	5′-CGTAGAGGTTGTACCAGCCG-3′	5′-TGAAGAGGAATGCCATGAACAAC-3′	5′-CTTCTCTCATCCTTGGCCACT-3′
*Lst Inx3*	5′-CGGTTATTACAACGTTCAATTAC-3′	5′-GCCAATGAGTACTTGAGAG-3′	5′-CTTGGTAGATGAACTTTTCCC-3′	5′-GGGAATGCTGTCATCCATTG-3′	5′-ATAGCTTACGTATGAACCAG-3′

**Figure 1:**
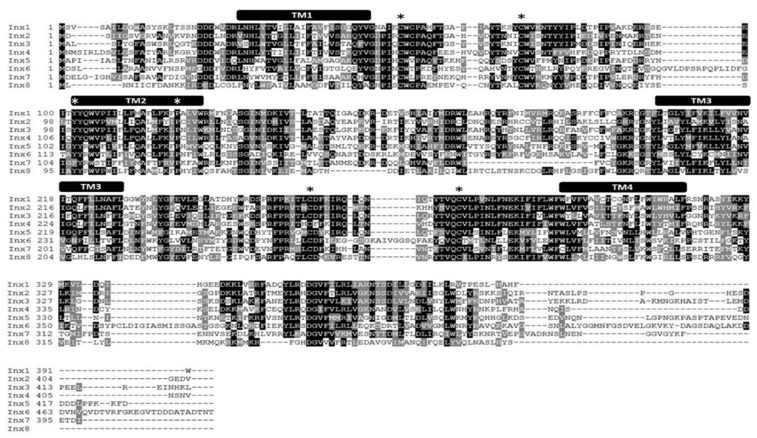
*L. stagnalis* express multiple paralogs of innexin in their central nervous system (CNS). RACE and PCR experiments revealed eight innexin paralogs within the *L. stagnalis* genome. All innexin sequences begin with a start codon and end with a stop codon. Amino acid alignment revealed conserved residues among the paralogs. Transmembrane domains are indicated above the sequences, and asterisks (*) indicate the two cysteines conserved across all innexins located in the two extracellular loops, the conserved YY(x)W motif, and the proline in the second transmembrane domain.

**Figure 2:**
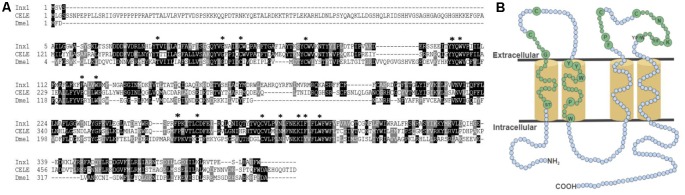
The conserved topology and sequences of innexins. **(A)** A multiple sequence alignment of *Lst Inx1* with one innexin in *C. elegans* and *D. melanogaster* shows the amino acid residues conserved in all innexins (shown in **B**). **(B)** In this model, cylinders are transmembrane domains, and circles represent amino acids. Small, blue circles signify a variation in number of amino acids while small, green circles signify an invariable number of amino acids. Residues conserved across all innexin sequences are written in big, green circles and are highlighted by an asterisk (*) in **(A)**.

To determine whether paralogous genes of *Lst Inx1* are present in the *L. stagnalis* genome, we then used the *Lst Inx1* amino acid sequence as a query to run a TBLASTN search against the genome sequence of *L. stagnalis* (assembly v1.0) from the NCBI WGS database (Skerrett and Williams, 2017). The BLAST search identified 10 significant hits (*E* value < 1e−10, alignment region <50% of query). Because the genome assembly of *L. stagnalis* is highly fragmented (328,378 scaffolds with N50 = 5,751), to determine whether each hit represented a unique genomic locus, we examined the genomic context for each hit. Three hits were found in three scaffolds that share 99.9% of sequence identities. The three hits were thus considered the same gene. Therefore, we identified eight paralogous genes of innexin in *L. stagnalis*, aptly named *Lst Inx1* through *Lst Inx8*. The nucleotide sequences of these innexin genes were translated to amino acid sequences *via* the ExPASy translation tool. 3′ and 5′ RACE (Table 3) was completed as previously described on *Lst Inx1*, and a complete open reading frame (ORF) from the start codon to the stop codon was obtained for a second innexin, *Lst Inx3*. The 3′ and 5′ ends of the remaining six genes were predicted *via* homology studies utilizing other invertebrate species’ innexin sequences including *B. glabrata* and *A. californica*. Three of the predicted genes, *Lst Inx2*, *Lst Inx5*, and *Lst Inx6*, were validated *via* PCR and primers designed in the first and last exons of each predicted sequence (Table 4). The eight sequences were then used in a multiple sequence alignment generated by T-Coffee (Figure 1), and a second multiple sequence alignment was generated with *Lst Inx1* and an innexin ortholog in *C. elegans* and *D. melanogaster*, CELE R07D5.1 and Dmel CG4590 INX2, respectively (Figure 2A; Notredame et al., 2000; Di Tommaso et al., 2011). Transmembrane domains were predicted with TMHMM Server v 2.0 software.

**Table 4:** Primers used for validation of predicted paralog sequences.

Paralog	Forward primer	Reverse primer
*Lst Inx2*	5′-CGTGAACCACCTGTACACCA-3′	5′-CTCTCCGTCACTCTCGTGTC-3′
*Lst Inx5*	5′-TGCATCACTGACCAACTTTGC-3′	5′-TGGTTCACGTCCTCACTGTC-3′
*Lst Inx6*	5′-GCTGCAGGAGTATGTTGGGAA-3′	5′-TTGGTATCTGCAGTGGCGTC-3′

### Phylogenetic Analysis

We used the amino acid sequence of *Lst Inx1* as a query to search for its homologous sequences *via* NCBI BLASTP in representative invertebrate species: the Molluscs *A. californica*, *Pomacea canaliculata*, and *Octopus bimaculoides*; the annelid species *Helobdella robusta*; the arthropod *D. melanogaster*; and the nematode *C. elegans*. A total of 89 innexin homologous genes were obtained from the seven species (*E* value < 1e−5, alignment region >50% of the query). The phylogenetic tree of the innexin gene family was inferred by using the maximum likelihood (ML) method implemented in MEGA 7 (Kumar et al., 2016). The best substitution model for the innexin sequences was inferred by the ML fit test tool in MEGA 7 (LG + G + I, *α* = 1.46).

### Tissue Expression Analysis of Innexins *via* Reverse Transcription (RT-PCR)

To identify expression of innexin paralogs across the body of *L. stagnalis*, RNA and gDNA were extracted from various tissues: CNS, buccal mass, penis, albumin gland, and foot. Seven ~12-month-old snails held in the same aquatic tank were chosen at random for dissection of tissue. The CNS was dissected from all seven snails, and other tissues were dissected from five of the snails. One CNS and two buccal mass samples were excluded from the experiment due to poor-quality RNA. RT-PCR was performed with SuperScript III One-Step Platinum Taq (Invitrogen; 12574-026; Carlsbad, CA, USA) and primers designed to amplify each paralog (Table 5). All gDNA extracted from the tissues underwent the same reactions for normalization; amount of template, primers used, and Mastercycler conditions were kept constant for all RNA and gDNA reactions. A 1% agarose gel was used to determine innexin paralog expression. All gel images were taken with the ChemiDoc MP Imaging System (Bio-Rad; 12003154; Hercules, CA, USA). Expected sizes of amplified products were *Lst Inx1* (498 bp), *Lst Inx2* (170 bp), *Lst Inx3* (404 bp), *Lst Inx4* (316 bp), *Lst Inx5* (147 bp), *Lst Inx6* (164 bp), *Lst Inx7* (446 bp), and *Lst Inx8* (404 bp). The intensity of expression (band intensity) was calculated with ImageJ software for both RNA and gDNA PCR products. The log2 ratio of the RNA-to-gDNA band intensity was calculated to represent relative gene expression as previously described (Tsankov et al., 2010). A no-RT control was used by heating the SuperScript III Platinum Taq mix at 95°C for 5 min to inactivate the enzyme according to the manufacturer’s instructions. A heatmap demonstrates the individual differences in relative innexin expression while a bar graph for each tissue type shows the average relative expression ± standard error of the mean (Figures 4A–F). Figures were created in R Studio.

**Table 5:** Primers used for tissue expression analysis.

Paralog	Forward primer	Reverse primer
*Lst Inx1*	5′-GTGGTTGGGCATCCTACTCC-3′	5′-ACTGCCTGTGGGCTTCTAAC-3′
*Lst Inx2*	5′-GGCAGATGACCAACAAGCAC-3′	5′-TATCCGAGACGACGGGAAGA-3′
*Lst Inx3*	5′-TGAAAGCCCATCGCCAGTAT-3′	5′-AACCGCAACCAGCAAATACC-3′
*Lst Inx4*	5′-ACGCTCGAGAGTACAGGTCT-3′	5′-GTGTAGTCGTGGACGTTGGT-3′
*Lst Inx5*	5′-GCTAAGCAGTACGTCGGTGA-3′	5′-ATCTGGGGCAAACGGTATGG-3′
*Lst Inx6*	5′-CGGCTGAAGATGGACGAAGT-3′	5′-TAGCACAGGTACAGGGACGA-3′
*Lst Inx7*	5′-GACTCTTGAGACCGCCAACA-3′	5′-TCCACTTGACGAGGCTGAAC-3′
*Lst Inx8*	5′-ACGGCCTGAGACACTTTCTG-3′	5′-GCGTATCCCCCACTTGAACA-3′

**Figure 3:**
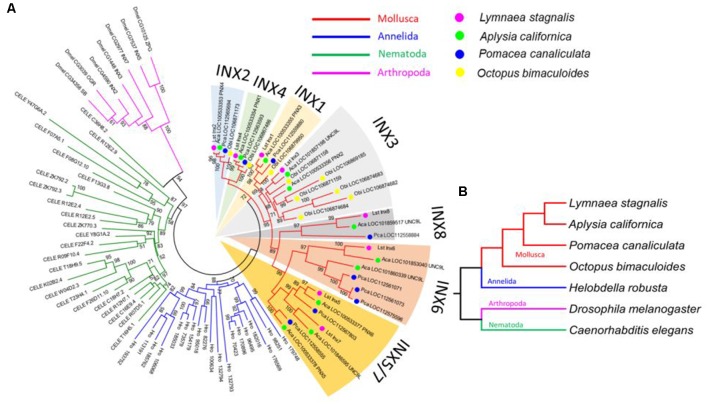
*L. stagnalis* innexins are evolutionarily related to innexins in other invertebrates. **(A)** Phylogenetic analysis revealed the evolutionary relationship between *L. stagnalis* innexin paralogs and innexin orthologs in other invertebrates including species within the Mollusca family, to which *L. stagnalis* belongs, and well-studied species within Annelida, Nematoda, and Arthropoda. The different families are separated by branch color: Mollusca (red), Annelida (blue), Nematoda (green), and Arthropoda (pink). The Mollusca family is further sorted by colored circles: *L. stagnalis* (pink), *A. californica* (green), *P. canaliculata* (blue), and *O. bimaculoides* (yellow). Shading is used to indicate the seven well-supported clades formed in Mollusca. **(B)** A phylogenetic tree demonstrates the evolutionary relationship between the species analyzed. The same branch color scheme is used as in **(A)**.

**Figure 4:**
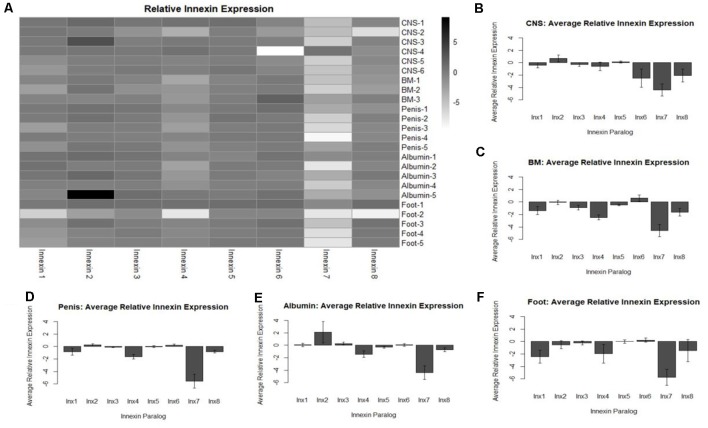
Analysis of relative innexin expression in different tissue types revealed both up- and down-regulation of innexin paralogs. RNA and gDNA from *L. stagnalis* was extracted from five tissue types and used in RT-PCR to determine relative innexin expression. **(A)** A heatmap shows variable expression between paralogs. One paralog, *Lst Inx7*, was down-regulated in most tissues with greater than 5-fold down-regulation in many cases. **(B–F)** Bar graphs demonstrate the average relative expression ± standard error of the mean for CNS **(B)**, buccal mass **(C)**, penis **(D)**, albumin gland **(E)**, and foot **(F)**. Sample sizes are as follows: CNS (*n* = 6), buccal mass (*n* = 3), penis (*n* = 5), albumin gland (*n* = 5), foot (*n* = 5).

### Quantification of Transcription Abundance of Innexin Genes Based on RNA-Sequencing Data

We downloaded the raw RNA-sequencing (RNA-seq) data of the CNS in *L. stagnalis* from the NCBI SRA database (SRA ID DRX001464). This dataset consists of 81.9 million single-end reads with a read length of 100 nucleotides. The RNA-seq reads were mapped to the genome sequence of *L. stagnalis* (assembly v1.0) using HISAT (Kim et al., 2015). Of these reads, 73.52% were aligned to the *L. stagnalis* genome exactly one time. The numbers of reads mapped to each innexin gene were counted by using the “featureCounts” (Liao et al., 2014). The transcription abundance of each innexin gene was normalized as reads per kilobase of transcript, per million mapped reads (RPKM, Supplementary Table S1).

### *In situ* Hybridization

**Figure 5:**
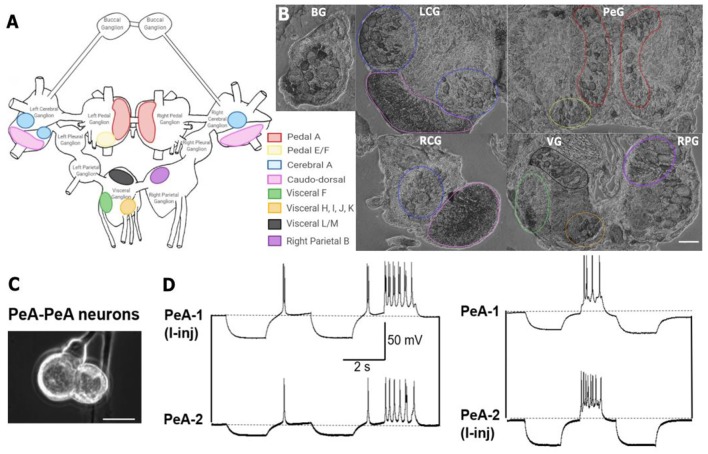
*In situ* hybridization with probes targeting *Lst Inx1* demonstrated localization of the transcript to neuron clusters throughout the *L. stagnalis* CNS. **(A)** A schematic of the *L. stagnalis* CNS shows the location of the eleven ganglia and highlights *Lst Inx1*-positive clusters. **(B)** ISH revealed localization of *Lst Inx1* mRNA in regions of specific ganglia, with colors in **(A)** corresponding to the same colored outline of clusters in **(B)**. Scalebar is 100 μm. **(C)** An example of individual neurons from *L. stagnalis* CNS that form functional gap junction coupling (electrical synapses) *in vitro*. **(D)** Intracellular recordings revealed that current injection (I-inj)-induced membrane potential change in one pedal dorsal A (PeA-1) neuron produces a synchronized membrane potential change in the paired PeA-2 neuron, and vice versa. Scale bar is 40 μm. Dotted lines indicate baseline membrane potentials. BG, buccal ganglion; LCG, left cerebral ganglion; RCG, right cerebral ganglion; PeG, pedal ganglion; VG, visceral ganglion; RPG, right parietal ganglion (*n* = 12 individuals).

To assess the localization pattern of *Lst Inx1* throughout the *L. stagnalis* CNS, ISH was performed with digoxigenin (DIG)-labeled probes. Twelve-month-old snails were randomly chosen and anesthetized, and the CNS was dissected. The commissure connecting the left and right cerebral ganglia was cut to allow the entire CNS to be splayed flat. Each CNS sample was paraffin embedded and sectioned into four ~10 μm slices. After sectioning, the samples were washed with xylene three times to dewax and rehydrated through an ethanol series (100% for two washes and 95%, 90%, 80%, 70%, and diH_2_O for one wash each). To allow hybridization, samples were fixed with 4% paraformaldehyde for 20 min and washed twice with DEPC-PBS for 5 min each. The samples were then treated with proteinase K (50 μg ml^−1^) at 37°C for 13 min. The samples were again washed in DEPC-PBS, fixed with 4% paraformaldehyde, and rinsed with DEPC-H_2_O. Pre-hybridization solution (BioChain; K2191050; Newark, CA, USA) was added to the samples for 4 h at 50°C followed by incubation in 4 ng ml^−1^ of DIG-labeled probe (Table 6) at 45°C overnight. Probes for *Lst Inx1* were designed to target regions with little sequence similarity between the eight paralogs, indicating the localization of the *Lst Inx1* transcript alone. Two probes were used against *Lst Inx1*, and the experiment was repeated three times with four snails each experiment to ensure reliability; one probe targeted the nucleotide 273 region while a second targeted the nucleotide 601 region with sense probes acting as controls. The samples were washed with 2× SSC, 1.5× SSC, and 0.2× SSC and incubated with blocking solution for 1 h at room temperature. To visualize the transcript, samples were incubated with AP-conjugated anti-DIG antibody for 4 h, washed with PBS and alkaline phosphatase buffer, and incubated with NBT and BCIP in alkaline phosphatase buffer overnight. After rinsing with diH_2_O, phase contrast images were taken on an inverted microscope (Olympus CKX53; Bridgeport, CT, USA). Images are shown in Figure 5.

**Table 6:** DIG-labeled probes used for *in situ* hybridization.

Transcript (nucleotide target)	Probe sequence
*Lst Inx1* (273)	5′-DIG-CACTGCTTCTCTCGTCCTTG-DIG-3′
*Lst Inx1* (601)	5′-DIG-ATGTACAGCCCGGTCAGAAA-DIG-3′

## Results

### Sequence Comparison of *L. stagnalis* Innexin Paralogs

RNA extracted from the CNS of *L. stagnalis* revealed eight paralogs of innexin, named *Lst Inx1* through *Lst Inx8*. The innexin sequences were transcribed and used to create a multiple sequence alignment with T-Coffee (Figure 1). A comparison of trends in the alignment and commonly conserved amino acids in innexins (Figure 2B) strengthened our confidence in the confirmed and predicted *L. stagnalis* sequences. For example, all invertebrate innexins share two strictly conserved cysteines in each extracellular loop and a YY(x)W region in the second transmembrane domain (Phelan and Starich, 2001); the eight innexin paralogs identified in *L. stagnalis* also shared these conserved regions. Topology studies with membrane-spanning protein prediction software revealed the expected four transmembrane structure of typical gap junction proteins in all the *L. stagnalis* innexins (Beyer and Berthoud, 2018). A separate multiple sequence alignment (Figure 2A) comparing *Lst Inx1* with the two most conserved innexin orthologs in *C. elegans* and *D. melanogaster*, CELE R07D5.1 and Dmel CG4590 INX2, respectively, revealed conserved amino acid residues common in innexins of all invertebrates (Figure 2B).

### Phylogenetic Analysis of the Origin and Evolution of Innexin Genes in *L. stagnalis*

To infer the origin and evolution of the eight innexin genes in *L. stagnalis*, we reconstructed a phylogenetic tree using the amino acid sequences from seven representative invertebrate species (“Materials and Methods” section; Figure 3A). The ML phylogenetic tree shows that all innexin genes from the four Mollusca species, namely, *L. stagnalis*, *A. californica*, *P. canaliculata*, and* O. bimaculoides*, form a well-supported monophyletic clade, suggesting a single origin of innexin genes in Mollusca. These Mollusca innexin genes form seven well-supported clades (Figure 3A, indicated by shaded regions), and each clade contains members from at least three Molluscan species. This topology suggests that multiple gene duplication events of innexin have occurred prior to the divergence of Mollusca, which generated at least five copies of innexin genes. One ancestral innexin gene was further duplicated before the divergence of *L. stagnalis*, *A. californica*, and *P. canaliculata* that generated *Lst Inx6–Lst Inx8*. Like Mollusca, innexins in other phyla, Annelida, Arthropoda, and Nematoda, also form a phylum-specific clade, suggesting that they originated from a single ancestral gene copy in each phylum followed by multiple gene duplication events (Figure 3B). The similar evolutionary patterns of innexin genes in major invertebrate phyla suggest that duplication and functional diversification of innexin genes might have played an important role in phylum-specific electrical synapse function and nervous system development.

### Expression of Innexin Throughout *L. stagnalis* Tissues

To gain a better understanding of the expression patterns of each innexin paralog throughout the body of *L. stagnalis*, we performed RT-PCR with primers specific to each paralog. Five organs of the snail were tested for specific reasons. The CNS was hypothesized to have very high levels of innexin expression. The buccal mass and foot are innervated by two sets of electrical synapse-forming cells, the octopamine neurons to regulate feeding and left/right pedal A neurons to regulate locomotion, respectively (Kyriakides et al., 1989; Vehovszky and Elliott, 2000). The albumin gland secretes epidermal growth factor required for synapse formation (Munno et al., 2000). The penis was used in a similar experiment testing the presence of nicotinic acetylcholine receptor expression (van Nierop et al., 2006) and was also used here. After RT-PCR with reactions using RNA or gDNA as starting material, agarose gel electrophoresis was completed (Supplementary Figure S2), with inactivated reverse-transcriptase reactions used as controls. Relative expression of each paralog was calculated throughout all tissues (“Materials and Methods” section, Figure 4). A heatmap was created to demonstrate the changes in paralog expression between individual tissue types (Figure 4A), and bar graphs show average relative expression for each tissue type tested (Figures 4B–F). Our results show that innexin genes are ubiquitously expressed throughout the entire body of *L. stagnalis*, and no obvious tissue-specific trends were imminent. However, innexin paralogs are upregulated and downregulated throughout the same tissue. For example, in sample CNS-1, *Lst Inx2* is upregulated while *Lst Inx7* is downregulated. Interestingly, *Lst Inx7* had noticeably less expression throughout all tissues tested. *Lst Inx4* also had generally lower expression in all tissue types while *Lst Inx2* had higher expression throughout the tissues.

### Localization of Innexin in the CNS

Previous work has identified many individual neurons in *L. stagnalis* CNS that form functional gap junctions (see Table 1). Because of this prior knowledge and because we found innexin paralogs could be expressed in the CNS (Figure 4), we next sought to determine innexin localization at the cellular level. To this end, probes targeting unique regions in the *Lst Inx1* sequence were employed in ISH (Figure 5) with sense probes used as a control (Supplementary Figure S3). ISH results showed differential localization of *Lst Inx1* within and between ganglia. For example, left and right pedal A (red) cluster neurons have relatively high *Lst Inx1* localization, mostly concentrated near the plasma membrane. Figures 5C,D shows sample recordings from our lab using *L. stagnalis* PeA neurons. Gap junction formation is revealed by current injection-induced change in membrane potentials in one cell causing synchronous changes in membrane potentials in its counterpart, confirming previous findings of gap junction-forming capabilities in pedal A neurons. Interestingly, a higher expression of transcript is localized to the left pedal ganglia than to the right pedal ganglia, indicating ganglionic heterogeneity in the expression of the same innexin paralog. Pedal E and F (yellow) and cerebral A (blue) cluster neurons also have high transcript localization. Similar heterogenic localization of *Lst Inx1* is found in the electrically coupled cerebral A cluster.

Prominent localization is found in the caudodorsal cluster neurons (pink), which are known to be electrically coupled to regulate ovulation hormone release (de Vlieger et al., 1980) and cells in the buccal ganglion that regulate feeding behavior (Benjamin and Rose, 1979). Transcripts are also localized to the cytoplasm of some cells of visceral F, H, I, J, and K clusters (green and orange). Visceral L/M (black) and right parietal B (purple) neurons demonstrate intriguing results; these clusters strongly localize *Lst Inx1* but have not yet been revealed electrophysiologically to be coupled. Electrophysiological experiments could support the hypothesis of electrical synapse abilities due to *Lst Inx1* localization.

Our results also demonstrate the lack of *Lst Inx1* localization on functionally defined electrical coupling cells. For example, the RPD2 cell, located in the right parietal ganglion, is known to form very strong electrical synapses with the ventral dorsal 1 (VD1) cell, located in the visceral ganglion (Söffe and Benjamin, 1980); however, neither cell localizes the *Lst Inx1* transcript. Perhaps, then, a different innexin paralog is being localized to permit strong electrical coupling in these cells. These results support the exciting possibility of a connection between cell specificity in innexin paralog localization and cell-specific functions. Overall, localization of *Lst Inx1* was distributed throughout the entire *L. stagnalis* CNS. Because ganglia localized *Lst Inx1* in some cells but not others, further study of the remaining innexin paralogs could determine if the cells undetected by *Lst Inx1* probe would localize a different innexin transcript.

## Discussion

Gap junction-mediated electrical synapses in the nervous system are ubiquitous throughout vertebrates and invertebrates (Stebbings et al., 2002; Nagy et al., 2018). They play essential roles in development and complex behaviors in all animals including humans. Invertebrate models such as *L. stagnalis* contain large and functionally identified gap junction-forming neurons and can be used as valuable resources to explore gap junction formation and channel gating mechanisms. The current lack of molecular information on gap junctions in *L. stagnalis* as well as in many other invertebrate systems, however, prevents a comprehensive understanding of gap junction formation, transmission, and plasticity. To address this significant knowledge gap, we, for the first time, identified and characterized the expression of gap junction genes in *L. stagnalis*. It is our hope that this original molecular work will bring more research avenues to the gap junction field using the robust model *L. stagnalis* for comparative physiology, fundamental neurobiology, and biomedical research. To this end, we identified eight innexin paralogs by initial sequencing and BLAST analysis against the *L. stagnalis* genome. The innexins showed similarity with other invertebrate innexins and exhibited the same topology as invertebrate innexins, vertebrate connexins, and pannexins (the vertebrate homologs of innexins; Baranova et al., 2004). Using RT-PCR and ISH, we provided evidence that innexin expression is paralog and ganglia specific, opening a potential link between innexin paralog expression and functional outcomes.

### Innexins in Invertebrates

Our study revealed at least eight innexin paralogs present in *L. stagnalis*, which are fewer than 25 paralogs in *C. elegans* (Altun et al., 2009) and 21 paralogs in *H. verbana* (Kandarian et al., 2012) but similar to eight paralogs in *D. melanogaster* (Stebbings et al., 2002), eight paralogs in *O. bimaculoides* (Albertin et al., 2015), six paralogs in *C. borealis*, and six paralogs in the lobster *Homarus americanus* (Shruti et al., 2014). In vertebrates, multiple paralogous connexins and pannexins are also seen; the human genome contains 20 connexins, the mouse genome contains 19 connexins, and both genomes contain three known pannexins (Eiberger et al., 2001; Baranova et al., 2004; Yen and Saier, 2007). An interesting question remains, then, as to the evolutionary significance of the existence of various numbers of gap junction genes in different organisms. In addition to the well-accepted reasoning that one paralog can compensate for the dysfunction of another paralog during gene loss or mutation-induced loss of function, evidence suggests the variation in connexin or pannexin gene number in vertebrates contributes to formation of heterotypic channels, leading to diverse channel functions, permeabilities, and gating mechanisms (Bukauskas and Verselis, 2004; Rackauskas et al., 2007). However, the physiological characteristics of diverse subunit combinations have yet to be fully explored in our and other invertebrate models.

The sequence alignment of innexin proteins revealed several interesting patterns among the eight *L. stagnalis* paralog sequences. For example, a proline residue located in the second transmembrane domain of all *L. stagnalis* innexins corresponds to the proline found in the same domain in connexins. In connexins, this proline may be involved in voltage gating-associated conformational changes (Sansom and Weinstein, 2000), an idea that has yet to be fully studied in invertebrates. Some obvious differences between *L. stagnalis* innexins were present at the amino- and carboxyl-termini, a common theme among gap junction sequences (Bauer et al., 2005). Structural work in *C. elegans* by Oshima et al. (2016) has suggested the involvement of the amino-terminus in the regulation of gap junction channel activity. In addition, the carboxyl-terminus was shown to determine the functional variability in connexins, as it is the site for modification *via* phosphorylation (Giepmans, 2004). As such, the differences in the amino- and carboxyl-termini of our *L. stagnalis* sequences suggest potential differences in functionality of the gap junction protein channels formed. Because *L. stagnalis* neurons are large and cell culture of coupled neurons has been well established (Syed et al., 1990; Feng et al., 1997; Xu et al., 2014; also see Figures 5C,D), an opportunity for combination of molecular and electrophysiological analysis in future studies is possible.

The multiple sequence alignment also revealed that *Lst Inx7* is the most divergent member. At the serine/threonine amino acid site in the first transmembrane domain (big, green circle in Figure 2B), *Lst Inx7* has a methionine. In the YY(x)W motif in the second transmembrane domain, *Lst Inx7* has a phenylalanine instead of a tyrosine at the first position. These amino acid residues are highly conserved among invertebrate innexins (Phelan and Starich, 2001). Therefore, the significant sequence divergence between *Lst Inx7* and other innexins is intriguing, and it is not known whether it is related to its low expression level (Figure 4, Supplementary Table S1). Nevertheless, it is interesting to note that one other innexin, *C. elegans Ce-inx-22*, also differs from typical innexin sequences at two amino acid residues: the first residue in the YY(x)W region in the second transmembrane domain and the proline position in the second extracellular loop (Phelan and Starich, 2001). In *C. elegans*, *Ce-inx-22* is expressed in germ cells, is thought to form heteromeric gap junctions with *Ce-inx-14*, and, along with *Ce-inx-14*, was screened as a negative regulator of oocyte maturation (Simonsen et al., 2014). An interesting future study could further explore the expression and role of *Lst Inx7* as a potential regulator of egg maturation to form a possible link between differences in amino acid residues and functional differences in paralogs.

### Evolutionary History of Gap Junctions in Invertebrates

Our phylogenetic analysis demonstrated that all eight innexin genes in *L. stagnalis* were generated before its divergence from *A. californica*. *A. californica*, like *L. stagnalis*, is a gastropod but is a saltwater slug while *L. stagnalis* is a freshwater snail (Moroz et al., 2006; Feng et al., 2009). *L. stagnalis* and *A. californica* diverged approximately 237 million years ago (Hedges et al., 2015). It would be interesting to learn why both species retained multiple paralogs of innexins. It is tempting to assume that the paralogous innexins in *L. stagnalis* and *A. californica* have experienced functional diversifications, resulting in different roles in the development and function of electrical synapses, and thus have been retained during the evolution of *L. stagnalis* and *A. californica*.

It is also interesting that all invertebrate phyla examined form phylum-specific clades and exhibit similar gene duplication patterns of innexins. Gene duplication is well accepted as a driving force of phenotypic evolution by generating raw genetic materials for functional innovation (Ohno, 1970). Functional innovation can be achieved by diversification of coding sequences and gene expression patterns (Zhang, 2003). The divergence of gene expression among innexin paralogous genes in *L. stagnalis* suggests that functional diversification occurred after the serial duplications of innexin genes during the evolution of *L. stagnalis*. The specific functions of each innexin gene in *L. stagnalis* still remain largely unclear. Future functional characterization of these innexin genes could provide further insight into understanding the evolutionary significance of innexins in invertebrates.

Another interesting observation is the lineage-specific expansion of innexin genes during the evolution of *O. bimaculoides*. *O. bimaculoides* lack members in clades of *Lst Inx5*, *Lst Inx6*, *Lst Inx7*, and *Lst Inx8*. Presumably, these results can be explained by two scenarios: *Lst Inx5*, *Lst Inx6*, *Lst Inx7*, and *Lst Inx8* and their related innexins were duplicated after the divergence of *O. bimaculoides*, or these genes have been lost in *O. bimaculoides*. Incorporating innexin in other species of octopus in future phylogenetic analyses would be interesting to determine if the *O. bimaculoides*-specific innexins are present in other octopus species. In addition, while the evolutionary history of innexins, connexins, and pannexins has been studied, the functional abilities of each channel are still unclear. For example, innexins in the leech *Hirudo medicinalis* can form gap junction channels, similar to connexins, as well as non-junctional channels, similar to pannexins (Bao et al., 2007). A phylogenetic analysis comparing innexins, connexins, and pannexins by functional ability could help explain why the three channel proteins have different functions.

### Paralog- and Ganglion-Specific Expression and Function

We wanted to delve further into the potential functional differences conferred by each *L. stagnalis* innexin paralog by first determining the paralog expression throughout tissue types. Our results demonstrated that all paralogs are expressed in every tissue but to different extents. In some cases, such as *Lst Inx4*, expression was highest in a single organ, the CNS. For *Lst Inx6*, though, the CNS had the least expression relative to other organs. This finding is not surprising considering the distribution pattern of gap junction genes in other invertebrates and vertebrates. For example, of the 21 innexins in *H. verbana*, only 11 are detectably expressed in the embryo CNS while five are expressed in the nephridia (Kandarian et al., 2012). The same variable gap junction gene distribution is found in human and mouse tissue (Oyamada et al., 2005).

Changes in gap junction expression are required for proper development, synaptic connections, and plasticity in invertebrates and vertebrates (Stebbings et al., 2002; Oyamada et al., 2005; Hall, 2017; Bhattacharya et al., 2019), and the differences in innexin expression levels found in *L. stagnalis* are likely required for proper organismal function under intrinsic, developmental and extrinsic, environmental regulations. Interestingly, *Lst Inx7* was downregulated compared to the other innexins in most tissues. This is consistent with the quantification analysis of previously published RNA-seq data showing that no reads mapped to *Lst Inx7* (Supplementary Table S1). However, it is important to note that this low expression shown in our study cannot exclude its expression and importance in other organs or under regulations by both developmental and environmental factors.

The effect of intrinsic and extrinsic factors on gap junction gene expression is an understudied field in invertebrates. Therefore, we wonder if other external or internal factors can help explain the differences seen in tissues of different snails. For example, hunger is known to change neuronal excitability in *L. stagnalis*. Dyakonova et al. (2015) found that the firing rate of electrical synapse-forming pedal A neurons significantly increased when *L. stagnalis* were deprived of food for 24 h. However, to our knowledge, no studies have looked at the effect of hunger on gene expression; a slight possibility exists that the number of days since the snails’ last feeding could affect the plasticity of electrical coupling and/or innexin expression. It would be very intriguing to test this postulation in future studies. In addition, an analysis of neuron-specific gene expression was recently completed in *C. elegans* which showed changes in innexin expression in response to transition to the dauer stage (Bhattacharya et al., 2019). Similar to *C. elegans*, knowledge of the expression patterns of innexin in *L. stagnalis* can be used to make strides in the understanding of how the electrical connectome is established throughout development and changes in response to external cues.

Finally, because we found evidence of potential paralog-specific functions at the tissue level, we wanted to establish if any differences also existed at the single-cell level. Because our RNA-seq analysis of transcription abundance of innexin genes showed *Lst Inx1* to be the most highly expressed innexin (Supplementary Table S1), we next performed ISH with DIG-labeled probes designed to target the *Lst Inx1* transcript. *L. stagnalis* CNS consists of relatively large neurons with well-studied neural networks. This was a significant advantage to our ISH study because we knew the ganglionic location and function of neurons with the ability to form electrical synapses. As mentioned previously, left and right pedal A cluster neurons are electrically coupled cells involved in pedal cilia used for locomotion (Kyriakides et al., 1989), and accordingly, our ISH data localized *Lst Inx1* in these neurons. We found ganglionic heterogeneity in these neurons as well as in cerebral A cluster neurons. In *A. californica*, intrinsic properties of individual neurons explained asymmetrical electrical coupling between neurons of the feeding motor network (Sasaki et al., 2013). In addition, in *L. stagnalis*, pedal A and cerebral A clusters form electrical synapses with cells in both their ipsilateral and contralateral counterparts (Kyriakides et al., 1989; Syed and Winlow, 1991). With these results in mind, the heteroganglionic localization of *Lst Inx1* could help explain the selective electrical connectome established in the pedal and cerebral ganglia. Likely, protein transcribed by *Lst Inx1* takes part in the formation of gap junctions in these ganglia; an antibody recognizing Lst Inx1 protein has the potential to confirm this hypothesis. If proven true, pedal A and cerebral A cells as well as other cells identified by this study to have *Lst Inx1* transcript localization could be used in further study of the voltage gating, permeability, and functional properties of the Lst Inx1 protein.

Localization of the innexin gene and protein paralogs is known to vary at the cellular level in invertebrates. In the CNS of *H. verbana*, innexins are expressed in a select number of cells. When ectopically expressed, though, *Hve-inx6* and *Hve-inx2* can form an electrical connection with cells to which they are not normally coupled. From these findings, the authors propose that the expression of a specific innexin paralog is sufficient for electrical coupling (Firme et al., 2012). In *C. elegans*, nearly every cell type expresses at least one paralog of innexin, allowing the formation of heterotypic and heteromeric gap junctions (Hall, 2017). Our results further support the theme of cell-specific innexin paralog expression and localization, which has potential for proper channel formation and changes in synaptic connection, ultimately leading to functional specificity of paralogs. Using the large, culturable neurons of our *L. stagnalis* model (Xu et al., 2014; also see Figures 5C,D) and the cell-specific innexin expression data we present here, pathways, mechanisms, and factors regulating electrical synapse formation and function can be studied in a novel light: the role of innexin genes in electrosynaptogenesis.

## Conclusion

Although electrical synapses were discovered in 1959, very limited information is available on the genetic aspects of gap junction formation and plasticity (Furshpan and Potter, 1959), which is mostly due to the extreme complexity of nervous systems in most model organisms. Discoveries made in animals with simpler nervous systems are, therefore, profoundly critical for the understanding of gap junctions in human and vertebrates. Importantly, recent studies have found that certain interactions and pathways involving innexins are conserved among vertebrates (Xu et al., 2001; Bauer et al., 2006; Alev et al., 2008; Welzel and Schuster, 2018). Considering the prevalent roles of electrical networks in nervous system development and function, characterizing the molecular underpinning of electrical synapses is urgently warranted. If detailed genetic information is available, powerful genetic tools allow an in-depth analysis and fine dissection of cellular pathways for understanding the basic mechanisms of physiology and pathology of gap junctions in our and other model organisms. Compared to the established models of *D. melanogaster* and *C. elegans*, *L. stagnalis*’ large neurons, functionally defined networks, and simple behaviors, together with its powerful synapse culture model and electrophysiology assays, provide a unique advantage to study the molecular, synaptic, and physiological mechanisms related to learning and memory as well as neurobiological diseases. The availability of *L. stagnalis* innexins provided by this study will aid our ability to study the molecular mechanisms related to gap junction formation and functions and eventually decipher their contribution to the physiology and pathophysiology of the nervous system. With our *L. stagnalis* model, we now have the means to answer specific questions, such as “What other transcription factors or proteins are used to regulate innexin expression and gap junction formation,” “Is there a compensatory mechanism used when one innexin paralog is inhibited, such as in a diseased state, to allow normal functioning,” and “Are these mechanisms and pathways conserved in vertebrate animals and humans?” Answers to these questions are critical to improve our understanding of the expression and function of gap junction genes and proteins, as well as inferring their evolutionary history and functional diversification in animals.

## Data Availability Statement

The datasets generated for this study can be found in the GenBank Accession Numbers: MN480796, MN480797, MN480798, MN480799, MN480800, MN480801, MN480802, MN480803.

## Author Contributions

BM, ZL, and FX designed the study. BM, SJ, and FX performed all experiments. BM and ZL performed data analysis and created the figures. BM and FX wrote the manuscript. BM, SJ, ZL, and FX revised and edited the manuscript.

## Conflict of Interest

The authors declare that the research was conducted in the absence of any commercial or financial relationships that could be construed as a potential conflict of interest.
