# Supplementary material for: Gap Junction Coding Innexin in Lymnaea stagnalis: Sequence Analysis and Characterization in Tissues and the Central Nervous System
Source: Front Synaptic Neurosci. 2020 Feb 25;12:1. doi: 10.3389/fnsyn.2020.00001 (PMC7052179; doi:10.3389/fnsyn.2020.00001)
Supplement: Supplementary file 1 [file Data_Sheet_1.pdf]

Supplementary Material

Supplementary Table 1. Transcription level of innexin genes of the CNS in *L. stagnalis* based on RNA-sequencing data

Gene	Transcript Length	Mapped Read Count	RPKM
Lst Inx1	1134	698	7.52
Lst Inx2	1065	11	0.13
Lst Inx3	921	108	1.43
Lst Inx4	855	188	2.68
Lst Inx5	990	131	1.62
Lst Inx6	1101	1	0.01
Lst Inx7	678	0	0
Lst Inx8	1209	0	0

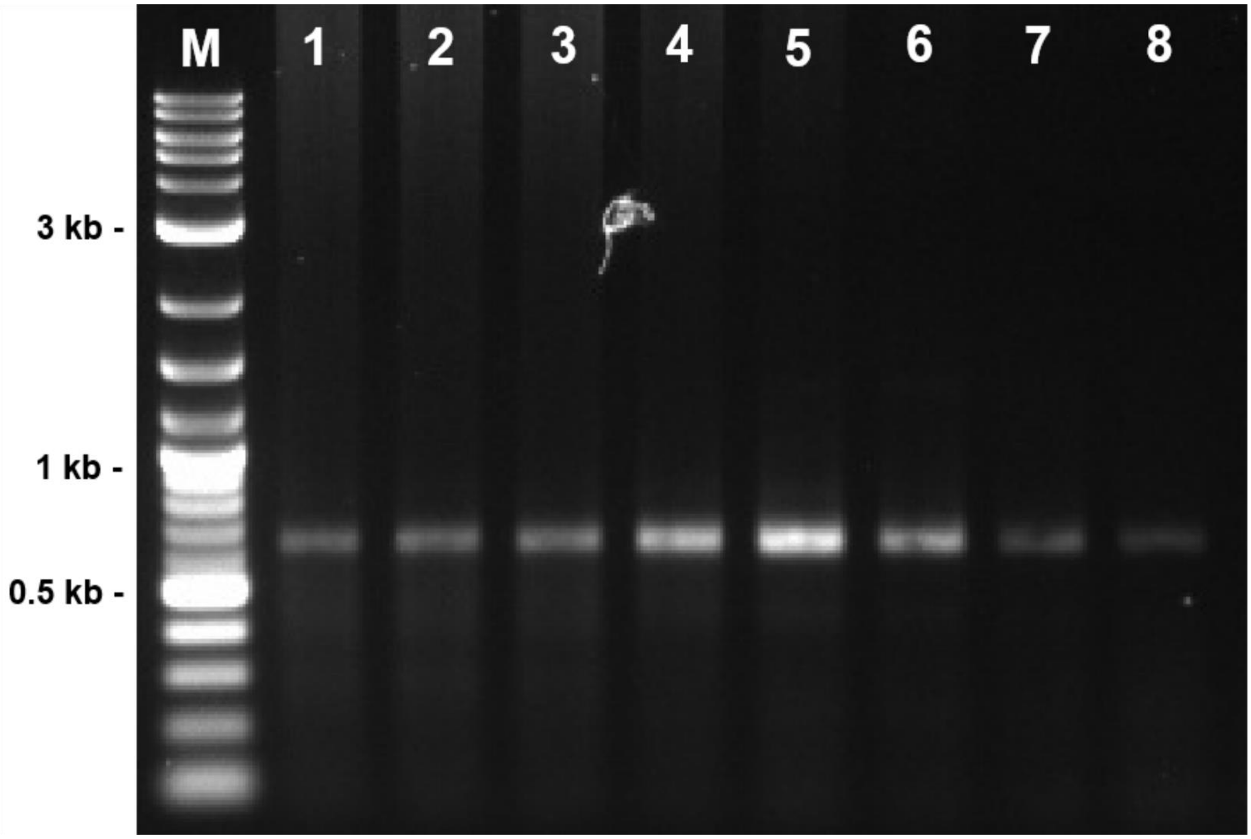

Supplementary Figure 1. PCR with degenerate primers created for *C. borealis* revealed a partial sequence of innexin in *L. stagnalis*. The CNS of 12 snails was dissected, and RNA was extracted. Annealing temperatures in PCR varied in lanes one through eight, but all settings resulted in the same-sized partial sequence: lane 1: 40.0˚C; lane 2: 40.2 ˚C; lane 3: 41.3˚C; lane 4: 43.1˚C; lane 5: 45.4˚C; lane 6: 48.0˚C; lane 7: 50.7˚C; lane 8: 53.5˚C. M=molecular ladder.

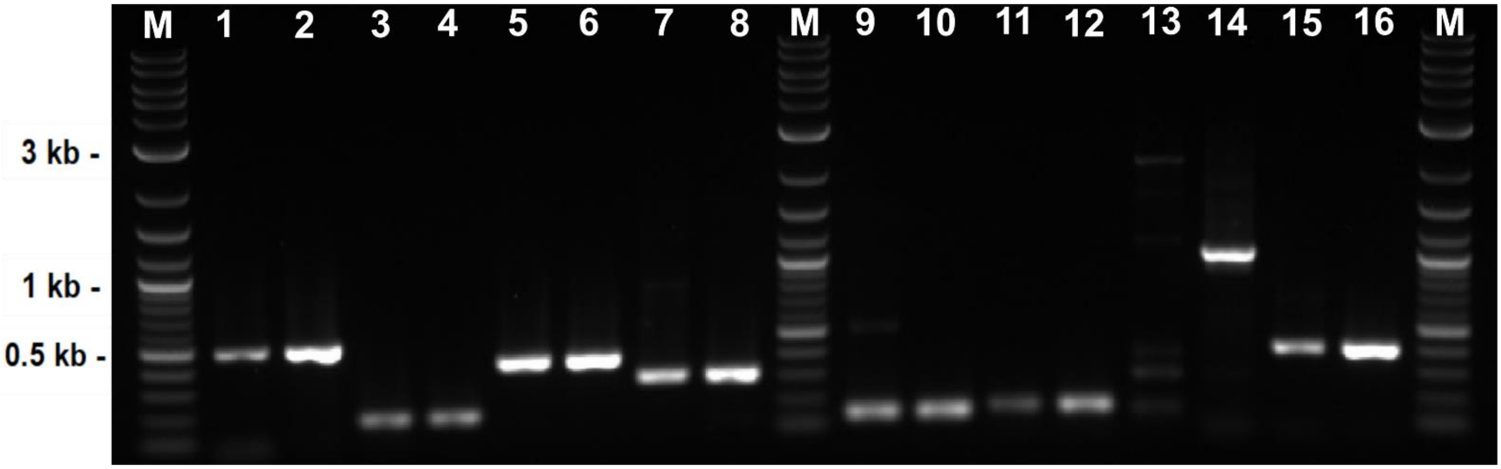

Supplementary Figure 2. Representative agarose gel shows the results of RT-PCR RNA and gDNA reactions. Here, RNA and gDNA was extracted from *L. stagnalis* CNS, and primers specific to each paralog were used (Table 5). Lane 1: *Lst Inx1* RNA; lane 2: *Lst Inx1* gDNA; lane 3: *Lst Inx2* RNA; lane 4: *Lst Inx2* gDNA; lane 5: *Lst Inx3* RNA; lane 6: *Lst Inx3* gDNA; lane 7: *Lst Inx4* RNA; lane 8: *Lst Inx4* gDNA; Lane 9: *Lst Inx5* RNA; lane 10: *Lst Inx5* gDNA; lane 11: *Lst Inx6* RNA; lane 12: *Lst Inx6* gDNA; lane 13: *Lst Inx7* RNA; lane 14: *Lst Inx7* gDNA; lane 15: *Lst Inx8* RNA; lane 16: *Lst Inx8* gDNA. Of note, the primer pair used for *Lst Inx7* is intron-spanning, and the resulting band includes one 558bp intron that is spliced out during mRNA production. Therefore, the *Lst Inx7* band in gDNA reactions is 1011bp rather than 446bp, like in RNA reactions. Designing an intron spanning set of primers added a level of control to the highly downregulated *Lst Inx7* to ensure the amplified RNA did not include any gDNA. The RNA and gDNA band intensities were used to create Fig. 4. M=molecular ladder.

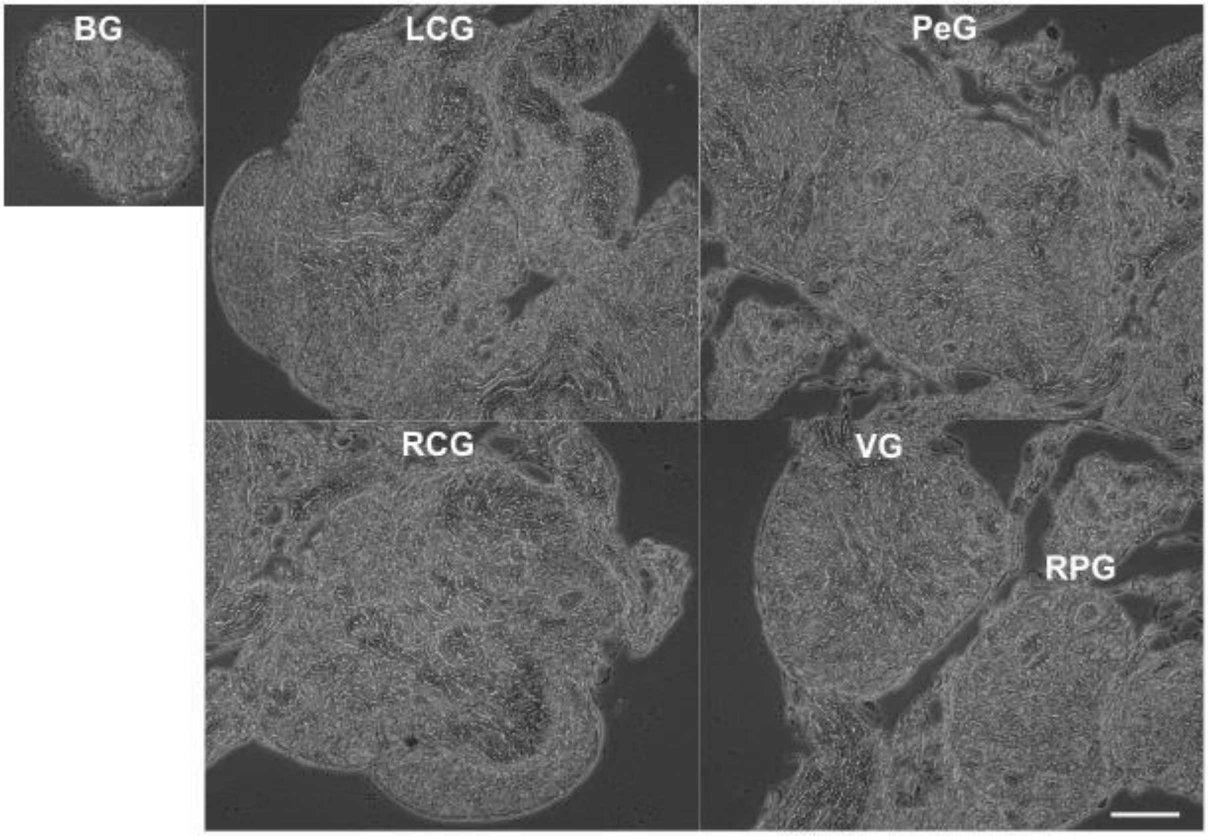

Supplementary Figure 3. Sense probes were used as controls for *in situ* hybridization. The same ganglia identified in Fig. 5 are shown here. BG: buccal ganglion, LCG: left cerebral ganglion, RCG: right cerebral ganglion, PeG: pedal ganglion, VG: visceral ganglion, RPG: right parietal ganglion. Scale bar is 100µm. (n=12 individuals)
